# Exploring Variation Between Artificial Grammar Learning Experiments: Outlining a Meta‐Analysis Approach

**DOI:** 10.1111/tops.12454

**Published:** 2019-09-08

**Authors:** Antony S. Trotter, Padraic Monaghan, Gabriël J. L. Beckers, Morten H. Christiansen

**Affiliations:** ^1^ Department of Speech, Hearing & Phonetic Sciences University College London; ^2^ Department of Psychology Lancaster University; ^3^ Department of English University of Amsterdam; ^4^ Department of Psychology, Cognitive Neurobiology and Helmholtz Institute Utrecht University; ^5^ Department of Psychology Cornell University; ^6^ Interacting Minds Centre and School of Communication and Culture Aarhus University; ^7^ Haskins Laboratories

**Keywords:** Artificial grammar learning, Meta‐analysis, Comparative studies, Visual modality, Auditory modality, Adjacent dependencies, Non‐adjacent dependencies

## Abstract

Artificial grammar learning (AGL) has become an important tool used to understand aspects of human language learning and whether the abilities underlying learning may be unique to humans or found in other species. Successful learning is typically assumed when human or animal participants are able to distinguish stimuli generated by the grammar from those that are not at a level better than chance. However, the question remains as to what subjects actually learn in these experiments. Previous studies of AGL have frequently introduced multiple potential contributors to performance in the training and testing stimuli, but meta‐analysis techniques now enable us to consider these multiple information sources for their contribution to learning—enabling intended and unintended structures to be assessed simultaneously. We present a blueprint for meta‐analysis approaches to appraise the effect of learning in human and other animal studies for a series of artificial grammar learning experiments, focusing on studies that examine auditory and visual modalities. We identify a series of variables that differ across these studies, focusing on both structural and surface properties of the grammar, and characteristics of training and test regimes, and provide a first step in assessing the relative contribution of these design features of artificial grammars as well as species‐specific effects for learning.

## Abstract 2

Studies of AGL have frequently used training and test stimuli that might provide multiple cues for learning, raising the question what subjects have actually learned. Using a selected subset of studies on humans and non‐human animals, Trotter et al. demonstrate how a meta‐analysis can be used to identify relevant experimental variables, providing a first step in asssessing the relative contribution of design features of grammars as well as of species‐specific effects on AGL.

## 1 Introduction

Artificial grammar learning (AGL) studies present learners with sequences of stimuli that inhere particular structural properties (Miller, 1958) of differing complexity (e.g., Reber, 1967), and then test learners on their ability to respond to sequences that incorporate aspects of this structure. Such an approach has been a very powerful method enabling investigations within a species into the possibilities and constraints on structural learning, such as distinctions between phrase–structure grammars or finite state grammars (e.g., Bahlmann, Schubotz, & Friederici, 2008), or the extent to which adjacent or non‐adjacent dependencies in sequences are available to the learner (e.g., Conway et al., 2010; Gomez & Gerken, 1999; Jamieson & Mewhort, 2005; Lai & Poletiek, 2011; Vuong, Meier & Christiansen, 2016). The paradigm is also of great potential use across species, and it has been extensively used to address questions about what structures are learnable by which species, and under what conditions (e.g., Abe & Watanabe, 2011; Chen et al., 2015; Fitch & Hauser, 2004; Saffran et al., 2008).

There has already been substantial progress made in addressing these questions, resulting in an intensive array of studies of learning in birds (e.g., Abe & Watanabe, 2011; Chen & ten Cate, 2015; Gentner et al., 2006; Spierings et al., 2015, 2017), non‐human primates (e.g., Endress et al., 2010; Heimbauer et al., 2018; Wilson, Smith, & Petkov, 2015), as well as human children and adults (e.g., Frost & Monaghan, 2017; Gomez & Gerken, 1999; Saffran et al., 2008), addressing acquisition of multiple grammatical structures across these species. The other papers in this special issue provide a host of further examples of the paradigm in use.

However, testing different structures and different species raises substantial methodological problems when it comes to direct comparisons between grammars and between species. Potential confounds both within and across studies have caused substantial concern in the past in terms of the validity of conclusions being drawn from studies (e.g., Beckers et al., 2012, 2017; Perruchet & Pacteau, 1990; Perruchet et al., 2004; de Vries et al., 2008), such as determining exactly what aspect of the structure is being responded to—whether that be the actual structures themselves, or some other feature of the stimuli (see, e.g., Knowlton & Squires, 1996). However, by using current meta‐analysis techniques, the presence of these potential confounds can actually provide valuable opportunities for teasing apart some of the multiple factors that may contribute to learning. Thus, the pattern of such confounds across studies provides a backdrop against which the contribution of specific experimental design decisions can be assessed in terms of their effect on participant learning. Critically, meta‐analysis permits researchers to quantify the effects of different kinds of stimuli within a species, but also differences across species in how they may respond to different grammatical structures. In this study, we present an analysis of a subset of AGL studies, providing a framework that more comprehensive analyses can follow.

In cross‐species comparisons, a key topic of interest is to determine which grammatical structures are potentially learnable by distinct species (Fitch & Friederici, 2012; Ghirlanda et al., 2017). The prospect of such discoveries has broad repercussions for the evolution of communicative systems, and the human specificity of language structure. The stakes are thus high. As one influential example, Fitch and Hauser (2004) conducted a study that required human adults and cotton‐top tamarins to distinguish between strings generated by a phrase–structure and a finite‐state grammar. Only Humans were able to make this distinction when trained on strings from the phrase–structure grammar. Subsequent research, however, has revealed several confounds in this study, suggesting that the humans may have relied on other sources of information to make their responses instead of the intended structural information (e.g. Perruchet & Rey, 2005; de Vries et al., 2008).

An ideal, perfectly controlled methodological study would isolate a particular grammatical structure and test learning of that particular structure without influence from other properties of the stimulus. However, the complexity of language structure and the practical challenges of training and testing different species on language‐like structures introduce variation into the actual tasks being conducted. Ensuring that only one particular aspect of language structure is tested, and tested in the same way across studies involving different species, remains a substantial, potentially insoluble, challenge.

In a recent small‐scale review of cross‐species studies of artificial grammar learning, Beckers et al. (2017) identified several characteristics that could have biased learning toward accepting the grammatical structure being tested without necessarily indicating learning of the structure. These included the extent to which the test sequence had previously occurred in the same form during exposure to the training sequences (either wholly or in part), whether the test sequence shared the same onset as the training sequences, and whether the test and training sequences were cross‐correlated even if they did not contain exactly the same sequences or subsequences. Thus, in a study containing one or more of these specific properties, it would be impossible to conclusively demonstrate that the grammatical rule was acquired by the learner. Such questions have been raised for almost as long as artificial grammar learning studies have been conducted—the extent to which learning is of particular grammatical structures or instead responding to lower‐level fragments in the sequences (cf. Knowlton & Squire, 1996; Perruchet & Pacteau, 1990—see Frost, Armstrong, Siegelman & Christiansen, 2015, for a review).

Artificial grammars also differ on fundamental structural properties. Some AGL studies contain dependencies between adjacent stimuli, whereas others contain dependencies between non‐adjacent elements in the stimuli. Furthermore, artificial grammars may differ in terms of the number of distinct stimulus elements that sequences contain, and the number of different categories to which these stimulus elements belong. An artificial grammar with a larger versus a smaller vocabulary, or a larger versus smaller set of grammatical categories, may affect learning distinctly. Learning studies can also vary in terms of the modality of the stimuli—whether they are auditory or visual (Heimbauer et al., 2018). For example, while cotton‐top tamarins are often trained on auditory (e.g. human non‐words, monkey calls; Neiworth et al., 2017) and visual materials (e.g. structured visuospatial sequences; Locurto, Fox, & Mazzella, 2015), zebra finches only receive auditory materials consisting of manipulations of species‐specific birdsong (e.g. Chen & ten Cate, 2015; van Heijningen et al., 2009). Modality is known to have distinctive effects on learning sequence structure (for reviews, see Frost et al., 2015; Milne, Wilson & Christiansen, 2018), and for these reasons modality is taken as a focus of the literature that we will analyze.

Artificial grammar learning studies also differ in terms of how training and testing is conducted. Studies of complex sequences with non‐human primates and birds may require substantial training time—several thousand trials over several weeks—whereas studies with human adults are typically constrained to short training sessions with a constrained set of training trials. Testing also varies in terms of how the effects of learning are measured. For instance, in testing human adults and children there is frequently a distinction between explicit, reflection‐based tasks for adult responses, such as alternative forced choice, or go/no–go responses, and implicit, processing‐based tasks such as head‐turn preferences or looking times. These tasks may tap into different mechanisms, with processing‐based tasks more effective for assessing processing‐based learning, such as acquisition of grammatical structures (Christiansen, 2019; Frizelle, O’Neill, & Bishop, 2017; Isbilen et al., 2018).

As we have summarized, studies of artificial grammar learning may vary along several of these dimensions simultaneously. In this paper, we present a blueprint for how a meta‐analysis approach could proceed to quantify how various design features of AGL studies might influence performance. We analyze a subset of AGL studies that have focused on presenting stimuli in either auditory or visual modalities, as reflected in the key words used within these articles. As we focus only on a subset of AGL studies, the conclusions drawn within the analysis may not generalize to the wider literature. The primary aim of our study is thus to provide a meta‐analytic framework that a more comprehensive study may adopt. We show how meta‐analytical methods enable us to measure the relative contributions of multiple potential confounds—reconsidered here as moderators—in influencing the size of the observed effects. This means that what was once considered a confound can actually be reinterpreted as providing a valuable and interesting source of data toward determining the limits and constraints on learning within and across species.

## 2 Method

### 2.1 Literature search

We conducted the literature search and meta‐analysis in accordance with the Preferred Reporting Items for Systematic Reviews and Meta‐Analyses (PRISMA) guidelines (Moher, Liberati, Tetzlaff, & Altman, 2009), pre‐registering the encoding and analysis to be conducted (https://aspredicted.org/wf2uk.pdf). The literature search was conducted on the SCOPUS database (Scopus, 2019) on articles published up to March 2019. In order to focus our literature review, we searched for studies that considered explicitly the modality of presentation in artificial grammar learning. We therefore conducted two searches of keywords appearing in titles, keywords, and abstracts of articles. In the first, we searched the keywords “artificial grammar learning” and “vision” OR “visual.” In the second, we used the keywords “artificial grammar learning” and “auditory” or “audio” or “audiovisual.” The results were then merged into a master list and submitted to study selection criteria.

The search we performed avoided bias in selecting publications for analysis, in accordance with PRISMA guidelines, but it is important to note that the results of the search were not comprehensive in including all papers that conducted AGL studies with auditory or visual stimuli. The literature search for instance failed to include several influential artificial grammar learning studies (e.g., Fitch & Hauser, 2004; Gentner et al., 2006; Reber, 1967; Saffran, 2001; Saffran et al., 2008). Our approach therefore outlines a blueprint for conducting meta‐analyses of potential design differences in AGL research, rather than to provide a final, comprehensive answer as to the size of effects of learning in AGL studies.

### 2.2 Study selection

**Figure 1:**
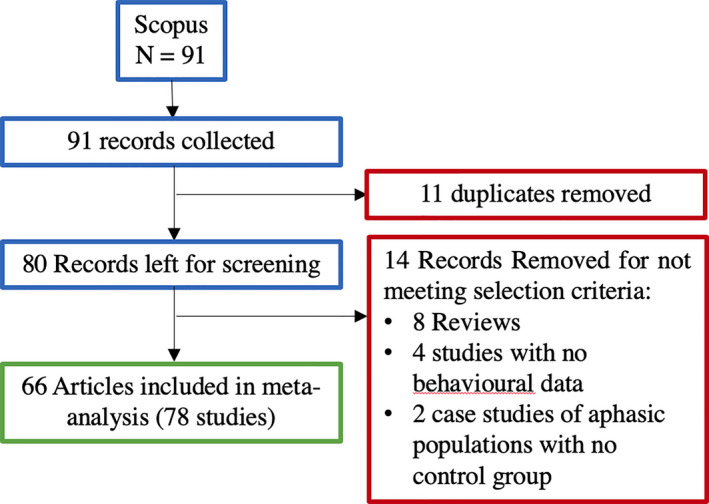
Flowchart of the PRISMA literature search criteria used in the current meta‐analysis.

The literature search resulted in 91 records. Of these, 11 were duplicates. Of the 80 articles remaining, 8 were review articles, 3 presented computational modeling and no behavioral data, 1 study reported neuroimaging data of primates with no behavioral data, and 2 reported a case study on an aphasic population with no control group. These articles were removed, and the remaining 66 articles contained 78 studies involving 3,559 subjects (this includes subjects tested more than once in the same article—see Results section for how the analysis took into account multiple studies within articles). Fig. 1 shows the PRISMA literature search flowchart. The list of studies included are reported in Data S1 and S2.

### 2.3 Data extraction and effect size calculation

The effect size for each study was initially computed as Cohen’s *d*, and subsequently corrected to Hedge’s *g*, with the variance of *g* computed in accordance with Borenstein et al. (2009). Formula (1) provides correction factor *J*, which is multiplied with Cohen’s *d* to provide Hedge’s *g* (2). The variance of Hedge’s *g*, *V_g_*, was provided by (3), where the variance of Cohen’s *d* is computed, and corrected by *J*.

(1) $$J=\left(1-\frac{3}{4df-1}\right),$$

(2) g=J×d,

(3) $$V_{g}=\left(\frac{1}{n}+\frac{d^{2}}{2\times n}\right)\times J^{2}.$$

Cohen’s *d* was derived for each type of dependent variable; the dependent variable for each study is shown in Data S1 and S2. For studies reporting the number correct, numbers endorsed or responded to, or go/no–go responses as the dependent variable, the effect size was computed from the difference to chance responding in a one‐sample test (see Eq. 4):

(4) $$d=\frac{Mean-Chance}{SD_{\mathrm{Within}}}.$$

In cases where tests and language structures were similar over different test sessions or conditions (e.g., Cope et al., 2017; Goranskaya et al., 2016; Mueller et al., 2010), we combined the means and *SD*s from each of the multiple test sessions, and computed the one sample difference from chance. The pooled mean was simply computed as the arithmetic mean across the sessions, weighted by number of participants in the session. For pooled *SD*, we took the average *SD* using Eq. 5, where *n*_1_ is the number of items in test session 1, *n*_2_ is the number of items in test session 2, etc., and *SD*_1_ is the observed standard deviation of the test session 1 response accuracy, etc. (see van Witteloostuijn, Boersma, Wijnen, & Rispens, 2017):

(5) $$SD_{\text{Average}}=\sqrt{\frac{\left(n_{1}-1\right)SD_{1}^{2}+\left(n_{2}-1\right)SD_{2}^{2}+\left(n_{3}-1\right)SD_{3}^{2}+\left(n_{4}-1\right)SD_{4}^{2}}{n_{1}+n_{2}+n_{3}+n_{4}-4}}$$

Subsequently, we computed *d* using Eq. 4, with the pooled mean, 50% as chance, divided by the *SD_Average_*. In serial reaction time studies, the effect was measured as the standardized mean difference in RT between presentations of a trained versus an untrained structure, with *SD*_Average_ computed as in Eq. 5, which assumes conservatively that there is a correlation of 1 between the trained and untrained structure responses across participants (a lower correlation would result in a lower *SD*, so this formula provides a conservative upper limit for the effect size). For instance, for Kemeny and Nemeth’s (2018) data represented in Fig. 3,we present the mean response time (RT) and *SEM* per testing block. In this case, we pooled the mean RT for the grammatical blocks 4 and 6 weighted by the number of participants in the session, and computed *d* as the difference to the mean RT for the ungrammatical block 5, with *SD* computed as the *SD*_Average_ across blocks 4, 5, and 6, using Eq. 5.

For sequence reproduction tasks, the effect size was computed as the difference in mean accuracy for grammatical sequences and ungrammatical sequences, with *SD* as the *SD*_Average_ computed using Eq. 5.

In head‐turn preference paradigms (e.g., Gomez & Gerken, 1999), effect size was the proportion of trials where the participant turned toward the grammatical violation sequences over the grammatical sequences, indicating observation of the violation. These values were compared to chance and *d* computed in the same way as for response accuracy measures.

For looking time paradigms (e.g., Milne et al., 2018), the effect size was computed as the difference in fixation duration between grammatical and ungrammatical sequences, computed using the same approach as that for sequence reproduction paradigms. Positive effects were generally computed as longer looking to ungrammatical than grammatical sequences (a novelty effect). However, in cases where the interpretation of the authors suggested that longer looking times to grammatical stimuli (or preferences in head‐turn to grammatical sequences) reflected greater learning (i.e., a familiarity effect), we re‐signed these effects.

In studies where means and variance were reported only in figures, we contacted authors for data and utilized the Digitizeit digitizer software (available from http://www.digitizeit.de/; Bormann, 2012) when such data were not available, to extract the means and *SD*s. In cases where graphs displayed the mean and 95% confidence intervals (Hall et al., 2018), confidence intervals were converted into *SD*s according to Eq. 6, which assumes that the authors had computed the confidence intervals using the *t*‐distribution (which is more conservative than assuming confidence intervals based on the *Z*‐distribution), where *tcrit* is the critical value of the *t*‐distribution for *n *− 1 degrees of freedom at *p* = .05:

(6) $$SD=\sqrt{n}\times \frac{upperlimit-lowerlimit}{2\times tcrit\left[n-1\right]}.$$

Each study was encoded for several features in order to test their influence on learning performance. We encoded the animal class and species that was tested, and in the case of human studies, distinguished whether the study was on children (<18 years) or adults.

For properties of the AGL structure, we encoded whether the study contained at least some repetitions of the stimuli experienced during training in the testing, whether the artificial grammar contained adjacent dependencies or did not contain adjacent dependencies, and whether the artificial grammar contained non‐adjacent dependencies or did not contain non‐adjacent dependencies.

For characteristics of training and testing, we encoded the type of test response that was being collected—whether this was a Yes versus No judgment, a go or no‐go task, a scale judgment, a forced choice test between two or more alternatives, serial reaction time, head‐turn preference, looking time, sequence production, or frequency estimation task. We subsequently grouped these variables into whether they required reflection on the grammatical structure (reflection‐based; forced choice tests, yes versus no judgment, go/no‐go, scale judgement), or more directly tapped into the underlying processing of the grammatical structure (processing‐based; looking time, head‐turn preference, serial reaction time, sequence production) (Christiansen, 2019). We encoded the amount of exposure to the artificial grammar that participants experienced in terms of the total number of stimulus tokens from the grammar during exposure (training length).

Importantly, we also encoded a number of surface features of the AGL, including whether the stimuli were visual, auditory, or a combination of both visual and auditory, in order to determine whether learning varied according to the modality of the task. Further, we also encoded the size of the artificial grammar in terms of the size of the vocabulary in the grammar (or the number of distinct items), as well as the number of different categories in the grammar (e.g., for a phrase–structure grammar with four nouns, two verbs, two adjectives, and two determiners, the number of categories is 4 (noun/verb/adjective/determiner) and the size of the vocabulary is 14.

## 3 Results

### 3.1 Evidence of acquisition of structure from AGL studies

The overall effect size across the studies, and the extent to which each of the encoded study variables predicted differences in effect sizes across the studies, was determined by conducting a random effects meta‐analysis of effect sizes, using the R package metafor (Viechtbauer, 2010). This approach takes into account inconsistencies between the studies analyzed, provides an estimate of sampling error, and also permits a measurement of the effects of each of the variables in moderating the size of the overall behavioral effect (Borenstein, Higgins, & Rothstein, 2009; Borenstein, Hedges, Higgins, & Rothstein, 2010). We encoded each experiment in an article and each test in an experiment as a separate study, and as these cannot be assumed to result in effect sizes independent from one another, we encoded the article as a nested multilevel variable in the analysis (Konstantopoulos, 2011).

**Figure 2:**
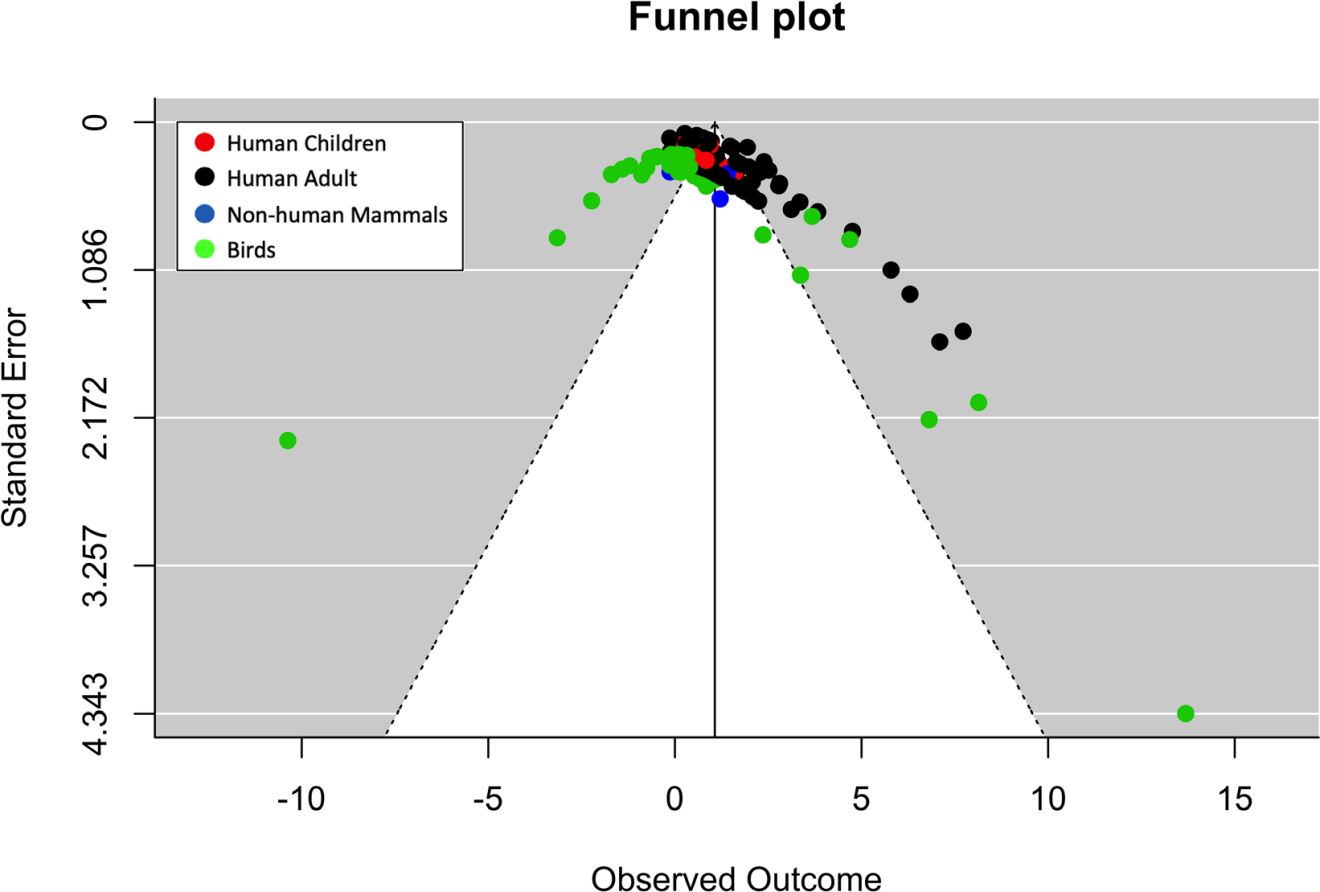
Funnel plot showing the relationship between the standard error and the effect size of the individual studies. Points are color‐coded according to animal class. Black points illustrate Human Adult Studies, blue illustrate Non‐human mammals studies, red are Human Child studies, and green are Bird studies.

The model was run using the rma.mv function with the restricted maximum likelihood (REML) method. We utilized the *t* method to generate test statistics and confidence intervals. The model was run using the rma.mv function with restricted likelihood (REML) method, and the *t*‐adjustment to calculate the model estimates of standard errors, *p* values, and confidence intervals. Effect sizes for individual studies and the overall average weighted effect sizes are presented in Fig. 2. A positive effect size indicates greater preference for stimuli conforming to the AGL structure, while a negative effect size indicates preference for non‐conforming stimuli (except in the case of the looking studies, where a positive effect indicates longer looking to violating stimuli—as this was the predicted effect of such studies in reflecting AGL acquisition, for example, Gomez & Gerken, 1999).

The meta‐analysis resulted in the average weighted effect size = 1.069 (*SE* = 0.130, 95% CI [0.813, 1.326], *p* < .0001), indicating that overall there was strong evidence of learning in AGL studies.

### 3.2 Publication bias

To determine whether there was publication bias in the sample, we conducted a Peters’ test (Peters et al., 2006) on the random multilevel meta‐regression model. The Peters’ test revealed a significant asymmetrical distribution, *t*(154) = −2.290, *p* = .023, indicating the presence of publication bias in our sample. The funnel plot (Fig. 2) displays the standard error (a measure of study precision) against the effect sizes of the individual studies. In the absence of publication bias, studies should be symmetrically distributed around the average weighted effect size in a funnel shape, with high precision studies being closer to the average weighted effect size, and lower precision studies symmetrically distributed around the average weighted effect size. The distribution indicates that there are more large positive effect sizes for smaller sample sizes than would be expected from a standard distribution of studies, suggesting a potential publication bias. The size of the effect of AGL acquisition, and the sources of heterogeneity of the effects, should thus be considered in light of possible bias in the studies published.

### 3.3 Heterogeneity in effect size variance associated with study variables

Cohran’s *Q*‐test for heterogeneity was significant (*Q*(155) = 1,185.657, *p* < .0001), indicating that variance in the data cannot be explained by random measurement error, but that different aspects of studies are contributing to the effect size. We thus analyzed the effects of each of the set of variables we encoded from each of the studies as moderators, shown in Table 1.

**Table 1:** Contributions of each moderating variable to account for variance in effect sizes across studies

Moderator	*F*	*Df1*,* Df2*	*p*
Population			
Animal species	2.613	(10, 145)	<.0001***
Animal class	5.811	(3, 152)	.0009***
Human vs. Non‐human	7.555	(2, 153)	.0007***
Training and testing			
Log training length	12.149	(1, 154)	<.0001***
Stimulus modality	0.095	(2, 153)	.909
Test response	1.624	(10, 145)	.105
Test type	3.698	(1, 154)	.056
Surface‐level properties			
Categories in language	0.0001	(1, 154)	.992
Number of unique vocabulary items	3.021	(1, 154)	.084
Structural properties			
Repetition of items	14.162	(1, 154)	.0002**
Adjacent dependencies	0.238	(1, 154)	.627
Non‐adjacent dependencies	0.118	(1, 154)	.608

Notes:

*F* is the statistic for testing whether the moderator accounts for some heterogeneity between studies; *p* is the significance for the *F‐*test ***p < .001, **p < .01, *p < .05. Note that Animal Class distinguishes birds, non‐human mammals, human adult, and human child. Animal species also distinguishes human adult and human child.

For the effect of animal class (but also distinguishing human adults and human children from non‐human mammals), there were significant differences on the size of effect of learning between different species. For human adults, the overall effect size was 1.252 (*SE* = 0.148, 95% CI [0.958, 1.545], *p* < .0001). For human children, the overall effect size was 0.615 (*SE* = 0.231, 95% CI [0.101, 1.129], *p* = .0237). For non‐human mammals, the overall effect size was 0.626 (*SE* = 0.172, 95% CI [0.221, 1.032], *p* = .008). For birds, the overall effect size was 0.428 (*SE* = 0.533, 95% CI [−0.653, 1.509], *p* = .427).

Properties of training and testing of AGL studies were found to produce significant differences in effect sizes. Log‐transformed number of training trials related negatively to effect size, −0.188 (*SE* = 0.054, 95% CI [−0.295, −0.0815], *p* = .0006). Further, repetition of trained items at test resulted in larger effects 1.051 (*SE* = 0.279, 95% CI [0.499, 1.602], *p* = .0002).

Surface‐level features of the language did not significantly moderate the variance of effect sizes (see Table 1), and this included also the modality of stimulus delivery. The number of categories, the vocabulary size, and critically, whether the stimuli were visual or auditory were not found to affect the overall effect size.

For the structural properties of the language, there were moderating effects. The presence of repetition of items from training to test positively influenced effect sizes, with an overall effect of 1.051 (*SE* = 0.279, 95% CI [0.499, 1.602], *p* = .0002).

As there were different sized effects of learning for each animal class, and possible confounds between study design characteristics and animal class tested, we conducted further analyses of moderator variables for human adult, human child, birds, and non‐human mammals separately.

### 3.4 Moderator analysis of human adults

There was significant heterogeneity of variance in the effect size in studies testing human adults (*Q*(99) = 707.273, *p* < .001), so we analyzed the effect of each moderator (see Table 2 for the significance of each moderator). There was a significant effect of the presence of non‐adjacent dependencies (effect = 0.582, *SE* = 0.259, 95% CI [0.068, 1.096], *p* = .027), suggesting that adult human participants are overall successful in learning non‐adjacencies in artificial grammars.

**Table 2:** Contributions of each moderating variable to account for variance in effect sizes in human adult studies

Moderator	*F*	*Df1*,* Df2*	*p*
Training and testing			
Log training length	0.415	(1, 98)	.521
Stimulus modality	0.306	(2, 97)	.737
Test response	0.671	(8, 91)	.716
Test type	1.884	(1, 98)	.173
Surface level properties			
Categories in language	0.319	(1, 98)	.574
Number of unique vocabulary items	1.023	(1, 98)	.305
Structural properties			
Repetition of items	0.036	(1, 98)	.851
Adjacent dependencies	1.745	(1, 98)	.190
Non‐adjacent dependencies	5.050	(1, 98)	.027*

Notes:

***p < .001, **p < .01, *p < .05.

### 3.5 Moderator analysis of human children

There was significant heterogeneity (*Q*(10) = 49.953, *p* < .0001), so we further analyzed the effect of each moderator (see Table 3). In this analysis, the only significant moderator was the test response participants made. This analysis indicated that head‐turn preference paradigms produced an overall effect of 1.301 (*SE* = 0.1663, 95% CI [0.772, 1.831], *p* = .004). Sequence production paradigms, by comparison, produced an effect that failed to statistically differ from 0 (effect size = 0.150, *SE* = 0.144, 95% CI [−0.433, 0.721], *p* = .395). Finally, binary yes–no judgement tasks produced an overall effect of 0.822 (*SE* = 0.099, 95% CI [0.506, 1.137], *p* = .004).

**Table 3:** Contributions of each moderating variable to account for variance in effect sizes in human child studies

Moderator	*F*	*Df1*,* Df2*	*p*
Training and testing			
Log training length	0.214	(1, 9)	.654
Stimulus modality	3.427	(1, 9)	.097
Test response	15.978	(2, 8)	.002**
Test type	0.271	(1, 9)	.615
Surface‐level properties			
Categories in language	0.059	(1, 9)	.813
Number of unique vocabulary items	0.862	(1, 9)	.377
Structural properties			
Repetition of items	2.503	(1, 9)	.148
Adjacent dependencies	0.023	(1, 9)	.884
Non‐adjacent dependencies	0.012	(1, 9)	.917

Notes:

***p < .001, **p < .01, *p < .05.

### 3.6 Moderator analysis of non‐human mammals

There was significant heterogeneity (*Q*(7) = 15.928, *p* < .026); therefore, we analyzed the effect of each moderator (see Table 4). Non‐human mammals only took part in studies delivered in the auditory modality, and all of which were processing based, included adjacent dependencies, and did not include repetitions at test, and hence we did not include a moderator analysis of testing modality, repetition of items, adjacency, and testing type. No moderator accounted for a significant proportion of variance in this dataset.

**Table 4:** Contributions of each moderating variable to account for variance in effect sizes in non‐human mammal studies

Moderator	*F*	*Df1*,* Df2*	*p*
Training and testing			
Log training length	1.121	(1, 6)	.331
Test response	1.262	(1, 6)	.304
Surface‐level properties			
Categories in language	0.760	(1, 6)	.418
Number of unique vocabulary items	0.365	(1, 6)	.567
Structural properties			
Non‐adjacent dependencies	0.111	(1, 6)	.750

### 3.7 Moderator analysis of birds studies

There was again significant heterogeneity (*Q*(36) = 259.498, *p* < .0001); therefore, we analyzed the effect of each moderator (see Table 5). Birds, however, only took part in classification‐based tasks, and thus, we did not analyze the effect of test type. Log training length accounted for a significant portion of the variance, and increased training resulted in a lower effect size −0.739 (*SE* = 0.268, 95% CI [−1.283, −0.195], *p* = .009). Increased vocabulary sizes tended to increase effect sizes (effect size = 0.099, *SE* = 0.038, 95% CI [0.022, 0.177], *p* = .014). Stimulus modality explained a significant portion of variance, with visual stimuli producing larger effects (effect size = 1.993, *SE* = 0.788, 95% CI [0.395, 3.592], *p* = .016) than auditory stimuli. The response task used also accounted for a significant portion of variance of effect sizes; however, the meta‐analytic estimate for both 2AFC tasks (effect size = 2.288, *SE* = 0.135, 95% CI [−0.488, 5.065], *p* = .090) and go/no‐go tasks (effect size = −0.042, *SE* = 0.294, 95% CI [−0.642, 0.559], *p* = .889) failed to significantly differ from 0. This reflects the fact that variance of effect sizes in birds was large; to properly account for the moderating effect of task type on the variance in effect size for bird studies, a larger set of studies for inclusion would be helpful. Finally, the repetition of items accounted for a significant portion of the variance of effect sizes, whereby repeating items at test resulted in an effect size of 5.013 (*SE* = 0.740, 95% CI [3.511, 6.515], *p* < .0001). This effect is explained by the only study including repetitions of whole strings at test (Spierings & ten Cate, 2016) produced large effect sizes.

**Table 5:** Contributions of each moderating variable to account for variance in effect sizes in bird studies

Moderator	*F*	*Df1*, *Df2*	*p*
Training and testing			
Log training length	7.609	(1, 35)	.009**
Stimulus modality	6.407	(1, 35)	.016*
Test response	6.407	(1, 35)	.016*
Surface‐level properties			
Categories in language	0.053	(1, 35)	.819
Number of unique vocabulary items	6.712	(1, 35)	.014*
Structural properties			
Repetition of items	45.926	(1, 35)	<.0001***
Adjacent dependencies	2.462	(1, 35)	.126
Non‐adjacent dependencies	1.661	(1, 35)	.206

Notes:

***p < .001, **p < .01, *p < .05.

## 4 Discussion

We presented a focused literature search analyzing AGL studies that address the modality of stimulus presentation, taking into account the varieties of designs, as well as species, that are tested across these studies. This approach provides a blueprint for how meta‐analysis in AGL studies can assess the influence of multiple moderators on learning, providing insight into the conditions under which learning of regularities in artificial grammars can be observed. Confounds and differences between studies—both intended and unintended (and previously viewed as adding opacity to the field of research)—can be considered sources of information for disentangling multiple contributors to learning of artificial grammar stimuli, rather than serve only as an impediment to comparison between studies. Heterogeneity of design can actually be analyzed through an estimate of heterogeneity of variance which can then be associated with the presence or absence of differences across studies.

This analysis was conducted to provide a framework as to how future, more comprehensive meta‐analyses might robustly identify patterns in the artificial grammar learning literature. However, our literature search was constrained by a restricted set of keywords that selected only papers where AGL and modality of presentation were explicitly tagged as features of the study. We know that influential studies in the literature were omitted by our approach. Whereas our focus here was to avoid bias in selecting the papers for inclusion in our analysis by conducting an objective keyword search, this absence of key studies highlights that there are relevant papers that are not included in the current analysis, and so the comprehensiveness of our search cannot be assumed. Consequently, the precise results of the meta‐analysis and the moderator analysis should not be taken as the final word on this topic. Instead, we have shown how a future analysis, on an even more comprehensive set of studies, may help move the field forward. Such a study will be a considerable undertaking; a Scopus search with the keywords “artificial grammar learning” or “statistical learning,” for instance, resulted in 6,511 records and still failed to include the landmark studies by Fitch and Hauser (2004), Gentner et al. (2006), and Reber (1967), mentioned in the Introduction, though the search did succeed in including the key studies by Saffran (2001) and Saffran et al. (2008). Finding principled ways to limit the literature search, without omitting key articles, presents an additional interesting challenge in this field of research.

This shortcoming raises concerns about terminological specificity in the field of artificial grammar learning. If we take Fitch and Hauser’s (2004) study, this paper explicitly implements an AGL method; however, it instead describes it as a “familiarization/discrimination paradigm” in its abstract. Gentner et al. (2006) do not describe their method in the abstract, and in text describe their method as a go/no‐go operant conditioning procedure of AB^n^ and A^n^B^n^ grammars. Similarly, Saffran’s (2001) and Saffran et al.’s (2008) methods are variously described as statistical learning, grammatical pattern learning, or familiarization–discrimination.

Cumming (2014) provided a compelling argument for favoring magnitude estimation over null hypothesis significance testing in assessing experimental effects. A tenet of this approach is to employ meta‐analytic thinking throughout the research process, including writing, reporting, and publication. The diversity of terms utilized to describe related methods makes it difficult to devise a singular, constrained set of search terms that would gather them together in a given search. Moving forward, we would suggest that using informative, umbrella keywords will ameliorate this issue, facilitating meta‐analyses, and in Cumming’s (2014) view, support research integrity.

In terms of the results of our focused meta‐analysis in terms of what can be learned across animal classes, the analyses showed that the size of learning effects varies according to the species tested, though the evidence of publication bias and the potential lack of comprehensiveness in the search mean that interpretations based on size of effects must be treated with caution. The overall largest effect was observed for studies involving adult humans, but there were also overall significant effects of learning associated with child humans, non‐human mammals, though not for birds. However, there are many differences between studies designed to appraise learning in different species, and heterogeneity of the variance within studies addressing each species points to ways in which these design differences may have profound effects on learning. The analyses of moderator effects within each animal class demonstrated that multiple variables were affecting learning, highlighting potential distinctions across species.

The size of the observed effects for human children was affected by the test response required, with similar effect sizes for head‐turn preference and Yes/No judgement tasks. While sequence production tasks did not significantly differ from 0, this likely reflects the small number of child studies included in the present analysis. For birds, the presence of training items at test produced large effects, perhaps unsurprising given the large amount of training they receive. Intriguingly, a greater number of training trials related negatively to effect size. This is likely correlated with the specific species of bird tested, and thus represents an important variable to focus on in a comprehensive meta‐analysis. For adult humans, larger effects were produced by grammars containing non‐adjacent dependencies than sequences without those dependencies, which have traditionally been difficult to observe in individual studies (e.g., Frost & Monaghan, 2016; Lai & Poletiek, 2011; Perruchet et al., 2004); see Wilson et al. 2019 in this issue for further discussion. The absence of a significant effect of adjacent dependencies was unexpected, but it highlights the variation that can occur in the effect sizes across studies testing these structures.

Further meta‐analytical techniques can help determine the additional sources of information that might support such learning, such as use of reflection‐ versus processing‐based test measures (Vuong et al., 2016). In order to measure the effect of learning on processing, rather than explicit decision‐making based on the structures experienced by the learner, a task that probes processing is proposed to be more effective (Christiansen, 2019; Frizelle et al., 2017; Isbilen et al., 2018); however, in the present analysis there was no statistically reliable difference between the two. This may be a consequence of the comparatively large number of reflection‐based effects (135) relative to processing‐based effects (21) included in this analysis, or of the range of grammars that tend to be tested in AGL studies, a large number of studies use Reber‐style (1967) grammars, where explicit testing may produce a similar magnitude of effects. Moreover, the effect of reflection‐based measures may also have been inflated by including the non‐human animal data as they are unlikely to engage in the kind of conscious reflections often observed in human studies. Finally, the presence of a potential publication bias combined with the much longer use of reflection‐based assessments in AGL studies going more than half a century may further explain this pattern.

A key issue that emerged during our analysis was that individual stimuli within a test may contain alternative structures or vary in the presence of surface features. The analyses in this paper report effect sizes and features of the stimuli across sets of stimuli, which can obscure the individual influence of these features. Making raw data sets publicly available would enable this by‐items analysis to reveal the precise contribution of multiple variables to learning behavior (e.g., Beckers et al., 2017).

The studies included here were selected from an objective literature search on SCOPUS, intending to avoid bias in our selection of tests, focusing on studies of AGL that describe the modality of the stimuli. Interestingly, except in the case of birds, modality was not found to affect the results, but this may also have been affected by observed publication bias. Expanding further to a literature search of an even broader literature would help to determine more clearly which moderators are affecting performance, and which are orthogonal to artificial grammatical learning. There are, for instance, other structures that are of key interest to both language acquisition research, and cross‐species investigations of the limits of grammar learning—such as distinctions between phrase structure and finite‐state grammars (Fitch & Friederici, 2012; Fitch & Hauser, 2004), or focused on hierarchical center‐embedded structures (Lai & Poletiek, 2011). Debates on the learnability of these structures (e.g., de Vries et al., 2008) will be facilitated by a wider survey of the published literature. In our blueprint for a meta‐analysis approach in this field, we have made an illustrative first step toward providing a perspective on what is learned and what is learnable within and across species.

## Supporting information


**Data S1:** List of studies included in the meta‐analysis.

**Data S2:** Meta‐analytical data
